# Longitudinal acquisition of endotracheal intubation skills in novice physicians

**DOI:** 10.1371/journal.pone.0188224

**Published:** 2017-11-14

**Authors:** Shinya Takeuchi, Takashi Shiga, Yasuaki Koyama, Taizo Nakanishi, Yosuke Honma, Hiroshi Morita, Tadahiro Goto

**Affiliations:** 1 Department of Emergency Medicine, Teikyo University, Itabashi, Japan; 2 Department of Emergency and Critical Care Medicine, Tokyo Bay Urayasu Ichikawa Medical Center, Urayasu, Japan; 3 Department of Emergency Medicine, International University of Health and Welfare, Minato, Japan; 4 Department of Emergency and Critical Care Medicine, University of Tsukuba Hospital, Tsukuba, Japan; 5 Department of Emergency Medicine, Fukui Prefectural Hospital, Fukui, Japan; 6 Department of Emergency Medicine, University of Fukui Hospital, Fukui, Japan; 7 Department of Emergency Medicine, Massachusetts General Hospital, Harvard Medical School, Boston, Massachusetts, United States of America; Waseda University, JAPAN

## Abstract

Little is known about the acquisition of intubation skills among novice physicians during their one-year clinical training. Our primary objective was to determine the changes in the intubation skills of novice physicians between prior to the clinical training and after completion of the clinical training. We used data of a prospective longitudinal multicenter data registry developed to investigate factors associated with the improvement of intubation skills among novice physicians. The study participants included 90 postgraduate year 1 physicians in 2015–2016. We used 4 simulation scenarios based on the devices used (direct laryngoscope [DL] and Airway scope [AWS]) and difficulty of intubation (normal and difficult scenarios). As a marker of the intubation skills, we used the force applied on the maxillary incisors and the tongue with each intubation. We compared the data obtained prior to clinical training with those obtained after completion of one-year clinical training. When using DL, compared to prior, significantly less force were applied on the maxillary incisors and the tongue after clinical training in the normal scenario (28.0 N vs 19.5 N, *p* < 0.001, and 11.1 N vs 8.4 N, *p* = 0.004). Likewise, when using AWS, compared to prior, significantly less force were applied on the tongue after clinical training in the normal scenario (22.0 N vs 0 N, *p* < 0.001). The force on the tongue decreased after clinical training but not significant. These associations persisted in the difficult airway scenario. These findings suggest that force applied on oral structures can be quantified as a marker of intubation skills by using high-fidelity simulators, and the assessment of procedural competency is recommended for all novice physicians prior to performing intubation in the clinical setting to improve the quality of emergency care.

## Introduction

Endotracheal intubation is an important skill for maintaining airway patency in the emergency department (ED). While intubation is one of the most frequently performed procedures in the ED, emergency intubation is associated with a high incidence of complications [1]. An observational study showed that 1.8% of emergency intubations had dental complications [2]. Additionally, several complications (e.g. bradycardia, cardiopulmonary arrest) are associated with the excess force applied on the oral structures [3–5]. In this context, intubation with a lower force applied on the oral structure is an important part of intubation skills to prevent complications in the ED.

In developed countries, 37%-50% of intubation procedures were performed by junior residents [6, 7]. Therefore, assessment of intubation skills in novice physicians should be warranted for patient safety and improving intubation outcomes. Although multiple studies have focused on the success of intubation during residency training [8, 9], there is a lack of research on whether the residency program and clinical training improve other important markers of intubation skills, such as force on the oral structures during intubation. The reasons of the scarce data include the difficulty of quantification of the force on oral structures and potential harm of on-site assessment of intubation skills in the clinical settings. Nevertheless, recent newly developed high-fidelity simulators with assessment systems enabled us to quantify the force applied on oral structures [10, 11].

In this context, we examined the differences in force applied on the oral structures (maxillary incisors and the tongue) during intubation performed by novice physicians between prior to clinical training and after completion of one-year clinical training.

## Methods

### Study design and settings

We prospectively collected and analyzed data from the Japanese Airway Management Quantification (JAMQ) study. The JAMQ study is a prospective multi-center data registry developed to investigate factors associated with the improvement of intubation skills among post-graduate year-1 (PGY-1) physicians using a high-fidelity simulator, with all data collection planned a priori [10–12]. The JAMQ study was initiated in April 2015 as a consortium of two academic hospitals (University of Fukui Hospital and University of Tsukuba Hospital), and two community medical centers (Fukui Prefectural Hospital and Tokyo Bay Urayasu/Ichikawa Medical Center), from different geographic regions across Japan. All enrolled subjects performed the simulation prior to their clinical training and again after completion of one-year clinical training using the same protocol. The present study utilizes the data collected in April 2015 and 2016, coinciding with a complete post-graduate training year. The PGY-1 physicians from four medical institutions were recruited for this study. All incomplete data were excluded. All participants provided written informed consent prior to participation in the study.

### Data collection

#### Simulation scenarios

We used data from four simulation scenarios that were based on the device used (the Macintosh Direct Laryngoscope [DL] or the Airway Scope [AWS] Video Laryngoscope) and the difficulty of intubation (normal and difficult scenarios). The simulation scenarios were as follows: (1) intubation of a normal airway using DL, (2) intubation of a normal airway using AWS, (3) intubation of a difficult airway using DL, and (4) intubation of a difficult airway using AWS. Size 3 and 4 laryngoscope blades were used for DL. We used a high-fidelity airway management simulator, Waseda Kyoto Airway No.5 (Kyoto-Kagaku, Kyoto, Japan), to quantify each participant’s intubation skills [10–12]. Implanted sensors in the simulator automatically quantified the force applied on the maxillary incisors and the tongue during an intubation attempt [10, 11]. We defined a ‘normal airway scenario’ as a scenario with a mouth opening to 4.5 cm. According to the previous study using the same simulator [11], we defined a ‘difficult airway scenario’ as a scenario with a limited mouth opening to 3 cm[12]. The limited mouth opening is a major cause of difficult intubation in the ED [13], thereby it is included in the modified LEMON criteria that are widely used to predict the intubation difficulty [14].

Prior to the simulations, all participants received a 15-min lecture and 15-min practice session to ensure familiarization with the proper techniques for DL and AWS. Following this training period, the participants were randomly assigned to one of the four simulation scenarios, and sequentially underwent the other simulation scenarios. All participants were blinded to the level of difficulty of each scenario.

#### Characteristics of participants

Information on participant demographics was collected, including sex, number of intubation-training courses attended, total number of intubation attempts, and number of intubation attempts made using AWS. Given that PGY-1 physicians have limited intubation experience, we included intubation attempts during the simulation training in the total number of intubation attempts. The number of intubation attempts was collected over the course of 12 months from each participant.

### Measurement of outcomes

Our primary outcome measures were the maximum force applied on the maxillary incisors and the tongue during intubation attempts, measured in Newtons (N). The secondary outcome measure was time to intubation, which we defined as the elapsed time from on-scene arrival (prepped for intubation) to successful placement of the endotracheal tube and confirmation of ventilation. An intubation attempt was defined as a single insertion of the laryngoscope (DL and AWS) past the teeth.

### Statistical analysis

Since the outcome variables were expected to have a non-normal distribution, we performed the Wilcoxon signed-rank test to compare the outcomes prior to their clinical training and after one-year of clinical training. P-values presented were from a two-tailed test, and values <0.05 were considered statistically significant. All data analyses were performed with EZR software V.1.28 (Saitama Medical Center, Jichi Medical University, Saitama, Japan), which is a graphical user interface for R (The R Foundation for Statistical Computing, Vienna, Austria). Specifically, it is a modified version of R commander designed to add statistical functions frequently used in biostatistics [15].

### Ethical issues

All subjects participated in this study voluntarily and provided written consent. This study was approved by the Institutional Review Board of University of Fukui, the University of Tsukuba Ethics Committee, the Ethics Committee of Fukui Prefectural Hospital, and the Ethics Committee of Tokyo Bay Urayasu/Ichikawa Medical Center.

## Results

Of 109 novice physicians (ie, PGY-1 physicians) identified at the beginning of our study, 19 were excluded due to incomplete data, and leaving 90 novice physicians were eligible for the analysis. Participant characteristics are shown in Table 1. Prior to clinical training, the overall median number of intubation attempts was 2 (interquartile range [IQR], 1–3), with a median of 0 (IQR, 0–1) with AWS application. After completion of the one-year clinical training, the median number of intubation attempts increased to 5 (IQR, 2–30), not including this trial.

**Table 1:** Baseline characteristics of participants.

Characteristics	(n = 90)
Men, n (%)	61 (68%)
Number of attendance of intubation training course prior to the clinical training, n (%)	
No attendance at an intubation training course,	36 (40%)
Attended 1 intubation training course,	34 (38%)
Attended 2 intubation training courses	18 (20%)
Attended 3 or more intubation training courses	2 (2%)
Total number of intubations prior to the clinical training^1^, median (IQR)	2 (1–3)
Total number of intubations using video laryngoscope prior to the clinical training, median (IQR)	0 (0–1)
Total number of intubations after completion of on-year clinical training, median (IQR)	5 (2–30)
Number of resident who had training in anesthesia after completion of one-year clinical training^2^, n (%)	17 (24%)
Number of attendance of intubation training course during one-year clinical training period^2^, n (%)	
No attendance at an intubation training course,	29 (41%)
Attended 1 intubation training course,	20 (29%)
Attended 2 intubation training courses	10 (14%)
Attended 3 or more intubation training courses	11 (16%)

IQR, interquartile range

^1^ Total number of endotracheal intubations corresponds to every participant's number of intubations over his or her career.

^2^ Participants data from University of Fukui Hospital and Fukui Prefectural Hospital were missing (n = 20).

When using DL, compared to prior, significantly less force was applied on the maxillary incisors during intubation in normal airway scenario after completion of one-year clinical training (median force [IQR]: 28.0 N [17.3–42.8 N] and 19.5 N [0–27.8 N], *p* < 0.001; Table 2). This association persisted in the difficult airway scenario (40.5 N [21.3–106.8 N] and 28.0 N [16.0–55.0 N], *p* = 0.002). With regard to the force applied on the tongue, compared to prior, significantly less force was applied on the tongue during intubation using DL in the normal scenario after completion of one-year clinical training (normal airway: 11.1 N [5.9–17.3 N] and 8.4 N [0.5–15.8 N], *p* = 0.004). Similarly, this association persisted in the difficult airway scenario (11.9 N [7.4–17.2 N] and 6.3 N [2.2–14.5 N], *p* = 0.001). In both scenarios, the median numbers of intubation attempts were 1 (both, IQR, 1–1).

**Table 2:** Comparison of force applied on oral structures and time to intubation, according the intubation devices and airway scenarios.

	DL			AWS		
	Prior to clinical training	After completion of one-year clinical training	p-value	Prior to clinical training	After completion of one-year clinical training	p-value
**Force on maxillary incisors (N)**						
Normal airway, median (IQR)	28.0 (17.3–42.8)	19.5 (0–27.8)	<0.001	22.0 (15.0–32.8)	0 (0–18.0)	<0.001
Difficult airway, median (IQR)	40.5 (21.3–106.8)	28.0 (16.0–55.0)	0.002	26.0 (19.3–37.0)	16.0 (0–21.8)	<0.001
**Force on tongue (N)**						
Normal airway, median (IQR)	11.1 (5.9–17.3)	8.4 (0.5–15.8)	0.004	1.1 (0–3.2)	0 (0–3.2)	0.46
Difficult airway, median (IQR)	11.9 (7.4–17.2)	6.3 (2.2–14.5)	0.001	1.1 (0–3.7)	0.3 (0–4.5)	0.10
**Time to endotracheal intubation (seconds)**						
Normal airway, median (IQR)	43.5 (37.8–74.8)	34.5 (27.0–45.9)	<0.001	58.5 (39.5–88.1)	28.1 (24.2–37.2)	<0.001
Difficult airway, median (IQR)	51.9 (38.5–72.2)	34.8 (28.5–43.7)	<0.001	72.4 (49.0–120.2)	30.5 (25.3–36.5)	<0.001

DL, Macintosh direct laryngoscope; AWS, Airway Scope; N, newton; IQR, interquartile range

When using AWS, compared to prior, the force applied on the maxillary incisors during intubation in the normal airway scenario after completion of one-year clinical training was significantly less (22.0 N [15.0–32.8 N] and 0 N [0.0–18.0 N]; *p* <0.001; Table 2). This association remained in the difficult airway scenario (26.0 N [19.3–37.0 N] and 16.0 N (0.0–21.8 N); *p* < 0.001). In both scenarios, the force applied on the tongue were decreased after completion of one-year clinical training but not significant. In both scenarios, the median number of intubation attempts was also 1 (IQR, 1–1).

The median times to intubation completion were improved across all strata. When using DL, compared to prior, median times to intubation completion after one-year clinical training were significantly less in both airway scenarios, (normal airway: 43.5 s [37.8–74.8 s] and 34.5 s [27.0–45.9 s]; difficult airway: 51.9 s [38.5–72.2 s] and 34.8s [28.5s–43.7s], respectively; both, *p* < 0.001). Likewise, when using AWS, compared to prior, median times to intubation completion were significantly less after completion of one-year clinical training in both airway scenarios (normal airway: 58.5 s [39.5–88.1 s] and 28.1 s [24.2–37.2 s]; difficult airway: 72.4 s [49.0–120.2 s] and 30.5 s [25.3–36.5 s], respectively; both, *p* < 0.001, both; Table 2).

## Discussion

In this multi-center longitudinal study, we found that, by using DL, novice physicians intubated with a less force on the maxillary incisors and the tongue after completion of one-year clinical training. We also found that, by using AWS, less force was applied on the maxillary incisors during intubation after completion of one-year clinical training, while the reduced force applied on the tongue was not statistically significant. These findings were consistent in both normal and difficult scenarios. Time to intubation was significantly shorter after completion of one-year clinical training across all strata. To our knowledge, this is the first study that demonstrates the improved force applied on the oral structures–an important part of intubation skills–after completion of one-year clinical training.

Our findings are consistent with the previous studies that reported the increasing experience is likely to have lower rate of intubation-related complications [16, 17]. In the previous large multicenter study of 4,094 patients, the intubation-related adverse event rate was 17% in PGY 1, 13% in PGY 2, and 10% in PGY 3 [16]. Additionally, in the field of critical care medicine, a previous study has shown that skill acquisition by residents can reduce the incidence of complications related to intubation [18]. Our observed findings corroborate with these prior findings and extend it by demonstrating the reduced force applied on the oral structures after completion of one-year clinical training.

Although the use of force applied on the oral structures are surrogates of intubation-related complications, there is a linkage between the force applied on the oral structures and intubation-related complications [19]. For example, excess force on the maxillary incisors increases the risk of dental injury—a frequent adverse event of intubation, and is the most common claim against intubators [20]. According to the prior study, force on the maxillary incisors greater than 150 N could be a risk factor for dental injury [19]. In the current study, 25% of novice physicians applied force of ≥100 N to maxillary incisors prior to the clinical training in this study; however, the force was significantly reduced after completion of one-year training. Therefore, our findings suggest that clinical training may reduce the incidence of dental trauma in the clinical settings.

Another linkage between the force applied on the oral structures and intubation-related complications is increased sympathetic activities due to the force applied oral structures (e.g., tongue). The increased sympathetic activities may cause hemodynamic responses, such as hypertension, tachycardia, and arrhythmia [17, 21, 22]. The plasma concentration of catecholamine increases in response to excess intubation stimulation [23]. Therefore, intubation should be performed with minimal force to reduce the number of adverse events. In this study, the force applied on the tongue decreased after one-year of clinical training. Thus, the clinical training of residents might reduce the risk of hemodynamic changes during intubation attempts.

When an intubator used AWS, the force applied on the tongue were decreased but not statistically significant due to the relatively small differences and the limited statistical power. Because AWS allows indirect visualization of the vocal cords and enables intubation without upward lifting force required to expose the glottis, intubators can intubate with less force on the tongue compared to intubation with DL, even if the intubator is a novice physician. Indeed, in the current study, the force applied on the tongue by using AWS (e.g., 1.1 N in the normal scenario) was relatively lower compared to those by using DL (e.g., 11.1 N in the normal scenario) at prior to clinical training. This is consistent with a prior literature that reported the association between the use of AWS and less force applied on the tongue compared to use of DL [10].

Our findings suggest that evaluating the intubation skills using a simulation-based assessment system may be useful to reduce the force applied on the tongue or incisors during intubation resulting in a decrease of intubation-related complications and improvement of quality of emergency care. More importantly, the use of simulator for the evaluation is harmless and repeatable. Although there are no well-defined “minimal” force applied on the oral structures to prevent complications, our results may suggest that novice physicians should intubate with less than 20 N for maxillary incisors and 10 N for tongue based on the force after completions of one-year training.

### Study limitations

We acknowledge potential limitations of our study. First, we quantified the force applied on the maxillary incisors and the tongue by using simulators. Therefore, this simulation-based study could not measure the complications itself. Nevertheless, previous studies have indicated the excess force is a risk of intubation-related complications [19]. Second, we did not calculate the sample size in advance, as this study was conducted retrospectively using a prospective multi-center data registry. However, the post hoc power calculation demonstrates that the power of our study was sufficient (power >0.90) for all primary outcomes examined. Third, our study was simulation-based; therefore, our findings may not necessarily reflect the outcomes in actual patients. For example, in clinical settings, various factors (e.g., cardiopulmonary resuscitation, bleeding, secretions) may interrupt intubation attempts and overall success of securing an airway. However, quantitative measurement of force on the oral structures during intubation is difficult in the clinical settings. Therefore, our observations, the quantitative measurements of intubation skills become the basis for the future investigation and education for emergency airway management. Lastly, this simulation-based study was performed in the controlled and safe environment (e.g. no noise, not an actual patient), thereby the suggested optimal force (≤20 N for maxillary incisors and ≤10 N for tongue) were applicable only to the simple situation and procedure.

## Conclusions

In this multi-center longitudinal study, among novice physicians, we found that the force applied on the oral structures were significantly improved after completion of one-year clinical training, suggesting that preferable force is ≤20 N for maxillary incisors and ≤10 N for tongue. In addition, the time to intubation was also improved after completion of one-year clinical training. These findings support the effectiveness of current residency program and clinical training to improve intubation skills. Furthermore, our observations also indicate that the assessment of intra-oral pressure monitoring using high fidelity simulators prior to intubation in the clinical setting is beneficial for resident physicians and students to potentially decrease dental and hemodynamic complications.

## References

[1] Kovacs G, Law JA, Ross J, Tallon J, MacQuarrie K, Petrie D, et al Acute airway management in the emergency department by non-anesthesiologists. Canadian journal of anaesthesia = Journal canadien d'anesthesie. 2004; 51(2): 174–180. doi: 10.1007/BF03018780 14766697 10.1007/BF03018780

[2] Wongyingsinn M, Songarj P, Assawinvinijkul T. A prospective observational study of tracheal intubation in an emergency department in a 2300-bed hospital of a developing country in a one-year period. Emerg Med J. 2009; 26(8): 604–608. doi: 10.1136/emj.2008.061192 19625561 10.1136/emj.2008.061192

[3] Dashti M, Amini S, Azarfarin R, Totonchi Z, Hatami M. Hemodynamic changes following endotracheal intubation with glidescope((R)) video-laryngoscope in patients with untreated hypertension. Research in cardiovascular medicine. 2014; 3(2): e17598 25478537 10.5812/cardiovascmed.17598 PMC4253794

[4] Lee H. The Pentax airway scope versus the Macintosh laryngoscope: Comparison of hemodynamic responses and concentrations of plasma norepinephrine to tracheal intubation. Korean journal of anesthesiology. 2013; 64(4): 315–320. doi: 10.4097/kjae.2013.64.4.315 23646240 10.4097/kjae.2013.64.4.315 PMC3640163

[5] Owen H, Waddell-Smith I. Dental trauma associated with anaesthesia. Anaesthesia and intensive care. 2000; 28(2): 133–145. 10788963 10.1177/0310057X0002800202

[6] Goto Y, Goto T, Hagiwara Y, Tsugawa Y, Watase H, Okamoto H, et al Techniques and outcomes of emergency airway management in Japan: An analysis of two multicentre prospective observational studies, 2010–2016. Resuscitation. 2017; 114: 14–20. doi: 10.1016/j.resuscitation.2017.02.009 28219617 10.1016/j.resuscitation.2017.02.009

[7] Kim C, Kang HG, Lim TH, Choi BY, Shin YJ, Choi HJ. What factors affect the success rate of the first attempt at endotracheal intubation in emergency departments? Emerg Med J. 2013; 30(11): 888–892. doi: 10.1136/emermed-2012-201708 23243044 10.1136/emermed-2012-201708

[8] Clark TR, Brizendine EJ, Milbrandt JC, Rodgers KG. Impact of an anesthesiology rotation on subsequent endotracheal intubation success. Journal of graduate medical education. 2013; 5(1): 70–73. doi: 10.4300/JGME-D-11-00268.1 24404230 10.4300/JGME-D-11-00268.1 PMC3613322

[9] Soleimanpour H, Gholipouri C, Panahi JR, Afhami MR, Ghafouri RR, Golzari SE, et al Role of anesthesiology curriculum in improving bag-mask ventilation and intubation success rates of emergency medicine residents: a prospective descriptive study. BMC Emerg Med. 2011; 11: 8 doi: 10.1186/1471-227X-11-8 21676271 10.1186/1471-227X-11-8 PMC3125215

[10] Nakanishi T, Shiga T, Homma Y, Koyama Y, Goto T. Comparison of the force applied on oral structures during intubation attempts by novice physicians between the Macintosh direct laryngoscope, Airway Scope and C-MAC PM: a high-fidelity simulator-based study. BMJ Open. 2016; 6(5): e011039 doi: 10.1136/bmjopen-2016-011039 27217284 10.1136/bmjopen-2016-011039 PMC4885424

[11] Goto T, Koyama Y, Kondo T, Tsugawa Y, Hasegawa K. A comparison of the force applied on oral structures during intubation attempts between the Pentax-AWS airwayscope and the Macintosh laryngoscope: a high-fidelity simulator-based study. BMJ Open. 2014; 4(10): e006416 doi: 10.1136/bmjopen-2014-006416 25296656 10.1136/bmjopen-2014-006416 PMC4194748

[12] Yohan N, Wang C, Tokumoto M, Solis J, Ishii H, Takanishi A, et al. Development of airway management training system WKA-4: provide useful feedback of trainee performance to trainee during airway management. International Conference on Complex Medical Engineering; 2012.

[13] Reed MJ, Dunn MJ, McKeown DW. Can an airway assessment score predict difficulty at intubation in the emergency department? Emerg Med J. 2005;22(2):99–102. doi: 10.1136/emj.2003.008771 15662057 10.1136/emj.2003.008771 PMC1726680

[14] Hagiwara Y, Watase H, Okamoto H, Goto T, Hasegawa K. Prospective validation of the modified LEMON criteria to predict difficult intubation in the ED. Am J Emerg Med. 2015;33(10):1492–1496. doi: 10.1016/j.ajem.2015.06.038 26166379 10.1016/j.ajem.2015.06.038

[15] Kanda Y. Investigation of the freely available easy-to-use software 'EZR' for medical statistics. Bone marrow transplantation. 2013; 48(3): 452–458. doi: 10.1038/bmt.2012.244 23208313 10.1038/bmt.2012.244 PMC3590441

[16] Goto Y, Watase H, Brown Calvin A. III, Tsuboi S, Kondo T, Brown David F. M., et al Emergency airway management by resident physicians in Japan: an analysis of multicentre prospective observational study. Acute Medicine & Surgery 2014(1): 8 Available from: http://onlinelibrary.wiley.com/doi/10.1002/ams2.43/pdf 10.1002/ams2.43 PMC5997229 29930851

[17] Kovac AL. Controlling the hemodynamic response to laryngoscopy and endotracheal intubation. Journal of clinical anesthesia. 1996; 8(1):63–79. 8695083 10.1016/0952-8180(95)00147-6

[18] Jaber S, Amraoui J, Lefrant JY, Arich C, Cohendy R, Landreau L, et al Clinical practice and risk factors for immediate complications of endotracheal intubation in the intensive care unit: a prospective, multiple-center study. Critical care medicine. 2006; 34(9): 2355–2361. doi: 10.1097/01.CCM.0000233879.58720.87 16850003 10.1097/01.CCM.0000233879.58720.87

[19] Quinn JB, Schultheis LW, Schumacher GE. A tooth broken after laryngoscopy: unlikely to be caused by the force applied by the anesthesiologist. Anesthesia and analgesia. 2005; 100(2): 594–596. doi: 10.1213/01.ANE.0000151390.71913.9B 15673900 10.1213/01.ANE.0000151390.71913.9B

[20] Gaudio RM, Feltracco P, Barbieri S, Tiano L, Alberti M, Delantone M, et al Traumatic dental injuries during anaesthesia: part I: clinical evaluation. Dental traumatology: official publication of International Association for Dental Traumatology. 2010; 26(6): 459–465. 21078071 10.1111/j.1600-9657.2010.00935.x

[21] Koyama Y, Nishihama M, Inagawa G, Kamiya Y, Miki T, Kurihara R, et al Comparison of haemodynamic responses to tracheal intubation using the Airway Scope((R)) and Macintosh laryngoscope in normotensive and hypertensive patients. Anaesthesia. 2011; 66(10): 895–900. doi: 10.1111/j.1365-2044.2011.06802.x 21770906 10.1111/j.1365-2044.2011.06802.x

[22] Nishikawa K, Matsuoka H, Saito S. Tracheal intubation with the PENTAX-AWS (airway scope) reduces changes of hemodynamic responses and bispectral index scores compared with the Macintosh laryngoscope. Journal of neurosurgical anesthesiology. 2009; 21(4): 292–296. doi: 10.1097/ANA.0b013e3181a9c6dc 19955890 10.1097/ANA.0b013e3181a9c6dc

[23] Derbyshire DR, Chmielewski A, Fell D, Vater M, Achola K, Smith G. Plasma catecholamine responses to tracheal intubation. British journal of anaesthesia. 1983; 55(9): 855–860. 6615672 10.1093/bja/55.9.855

